# Sex differences in the extent of acute axonal pathologies after experimental concussion

**DOI:** 10.1007/s00401-024-02735-9

**Published:** 2024-05-05

**Authors:** Hailong Song, Alexandra Tomasevich, Andrew Paolini, Kevin D. Browne, Kathryn L. Wofford, Brian Kelley, Eashwar Kantemneni, Justin Kennedy, Yue Qiu, Andrea L. C. Schneider, Jean-Pierre Dolle, D. Kacy Cullen, Douglas H. Smith

**Affiliations:** 1 Department of Neurosurgery, Center for Brain Injury and Repair, University of Pennsylvania, 3320 Smith Walk, 105 Hayden Hall, Philadelphia, PA 19104 USA; 2 Center for Neurotrauma, Neurodegeneration and Restoration, Michael J. Crescenz Veterans Affairs Medical Center, Philadelphia, PA 19104 USA; 3 Department of Bioengineering, School of Engineering and Applied Science, University of Pennsylvania, Philadelphia, PA 19104 USA; 4 Department of Neurology, University of Pennsylvania, Philadelphia, PA 19104 USA; 5 Department of Epidemiology, Biostatistics, and Informatics, University of Pennsylvania, Philadelphia, PA 19104 USA

**Keywords:** Concussion, Sex difference, Diffuse axonal injury, Amyloid precursor protein, Voltage-gated sodium channel isoform 1.6, Axon ultrastructure

## Abstract

Although human females appear be at a higher risk of concussion and suffer worse outcomes than males, underlying mechanisms remain unclear. With increasing recognition that damage to white matter axons is a key pathologic substrate of concussion, we used a clinically relevant swine model of concussion to explore potential sex differences in the extent of axonal pathologies. At 24 h post-injury, female swine displayed a greater number of swollen axonal profiles and more widespread loss of axonal sodium channels than males. Axon degeneration for both sexes appeared to be related to individual axon architecture, reflected by a selective loss of small caliber axons after concussion. However, female brains had a higher percentage of small caliber axons, leading to more extensive axon loss after injury compared to males. Accordingly, sexual dimorphism in axonal size is associated with more extensive axonal pathology in females after concussion, which may contribute to worse outcomes.

**Supplementary Information:**

The online version contains supplementary material available at 10.1007/s00401-024-02735-9.

## Introduction

Each year, approximately 50 million individuals world-wide suffer a concussion, also commonly referred to as mild traumatic brain injury (TBI) [32]. However, there is nothing ‘mild’ about this condition for the more than 15% of individuals who suffer persisting neurocognitive dysfunction [34, 36]. There is mounting consensus that concussion induces acute structural and physiologic disruption of brain-network connectivity and function. In particular, the multifocal appearance of axonal pathology across the white matter, generally referred to as diffuse axonal injury (DAI) [1, 47, 51], is thought to be an important pathologic substrate underlying the clinical manifestations of concussion [5, 6, 25, 27, 33, 37, 44, 51].

While post-mortem examination of concussion in humans is limited due to its generally non-lethal nature, one study of six individuals who died shortly after injury due to other causes has identified DAI as the only neuropathological change [6]. The principal mechanical force associated with the induction of DAI is head rotational acceleration [35]. While these forces induce dynamic mechanical deformation of tissue across the brain, white matter axons appear selectively vulnerable to mechanical damage. This is due in part to their very thin, delicate, and elongated architecture and high anisotropic organization in white matter tracts. Indeed, dynamic deformation of axons induces immediate mechanical breaking of axonal microtubules [54], thereby inducing axonal transport interruption and accumulation of proteins in hallmark periodic swellings along the axon, which historically have been considered the morphologic signature of DAI [20, 25]. However, DAI may represent a spectrum of axonal injury phenotypes [27, 51]. Using a clinically relevant swine model of concussion via head rotational acceleration biomechanically scaled to human concussion [8, 14, 18], we recently also observed disruption of axonal sodium channels (NaChs) across the white matter, similar to axonal NaCh loss we identified in higher severities of TBI in humans [51]. Collectively, these acute pathologic processes of DAI are thought to play key roles in the physical and functional loss of brain connectivity after concussion.

Although males dominate emergency department visits for concussion, this has been primarily attributed to their greater exposure to activities with a risk of head impacts compared to females [19]. In contrast, it has recently been observed that female athletes have a higher rate of concussion and appear to have worse outcomes than their male counterparts participating in the same sport [4, 7, 13, 53]. While many factors are likely at play here, this raises the intriguing possibility that sex-based differences in the extent of axonal pathology could contribute to differences in concussion outcome, potentially related to sex differences in average axon size and architecture.

It is long proposed that axons in the corpus callosum of human females might contain a greater percentage of small diameter axons compared to males [58], which has now been demonstrated by direct measurement in post-mortem microscopy examinations [39]. In addition, magnetic resonance imaging (MRI) studies have also shown smaller white matter volumes in human females than in males [21, 31]. Since female brains are approximately 8–13% smaller than male brains [41], it has been suggested that by having a reduced axon size, females can accommodate a similar number of axons forming the brain’s networks as males. Recently, we found that sexual dimorphism of axon structure also appears in both rat and human-induced pluripotent stem cell (iPSC) neurons in vitro [16]. Further, using an in vitro model that is based on concussion biomechanics [16], dynamic stretch injury of micropatterned unmyelinated axonal tracts induced greater microtubule damage and ionic disruption in female axons compared to male axons under the same level of injury. However, it has not been known if this link between potential sex differences in axon architecture and extent of axonal pathogenesis might also occur in vivo in brain myelinated axons due to concussion.

Here, using a well-characterized swine model of concussion [9, 14, 27, 28, 51], we explored potential sex differences in the extent of axonal pathologies in relation to axon size. Scaled to closely mimic the head rotational acceleration kinematics of human concussion, this model induces selective axonal pathologies in the absence of neuron death or gross pathologic changes [9, 14, 27, 28, 51]. We first examined the extent of axonal pathology at 24 h post-injury using the ‘gold standard’ immunohistochemical (IHC) staining for amyloid precursor protein (APP), which identifies periodic axonal swellings that arise due to microtubule damage and impaired axonal transport [20]. In addition, we examined the extent of loss of the predominant axonal NaCh that populates the nodes of Ranvier (NOR), Nav1.6 [51]. Moreover, using transmission electron microscopy (TEM), we evaluated potential differences in axonal diameter between female and males with no injury and changes in the average caliber of white matter axons after injury between the sexes.

## Materials and methods

### Experimental design and the swine head rotational acceleration model of concussion

Due to their relatively large gyrencephalic brain with extensive white matter, swine are ideally suited to model concussion to be clinically relevant to human concussion [14]. A total of 16 swine (Hanford strain, Sinclair Research Center, Inc.), aged approximately 6 to 8 months, were used and randomly assigned into four groups: sham female (*F*) (*n* = 3), sham male (*M*) (*n* = 3), injury F (*n* = 5), and injury-M (*n* = 5). All detailed animal characteristics, including age, body weight, brain mass, and injury level (maximum angular velocity) were listed in Table 1. For the injury group, swine were subjected to experimental concussion via rapid head rotational acceleration scaled to closely mimic human concussion biomechanics [8, 9, 14, 18, 27, 28, 51]. Extensive previous characterization demonstrated that this rotational acceleration resulted in selective axonal pathologies, which is morphologically identical to that observed in human [6, 20, 27, 51]. In addition, in this model, axonal pathology is seen in the absence of other pathologic features, e.g., hemorrhage, raised intracranial pressure (ICP), brain swelling or neuron death [14, 27, 28].

**Table 1:** Animal characteristics, injury kinematics, and recovery duration

		Sham-F (*n* = 3)	Sham-M (*n* = 3)	Injury-F (*n* = 5)	Injury-M (*n* = 5)
Age (months)	Mean (range) ± SD	6.2 (6.1–6.3) ± 0.1	6.3 (6.1–6.5) ± 0.2	6.6 (5.9–7.4) ± 0.7	7.1 (6.2–7.9) ± 0.6
Body weight (kg)	Mean (range) ± SD	36.9 (34.0–41.5) ± 4.0	34.1 (31.2–37.2) ± 3.0	29.2 (25.0–35.4) ± 4.0	36.1 (29.4–43.0) ± 4.9
Brain mass (g)	Mean (range) ± SD	98.4 (90.1–111.3) ± 11.3	96.7 (91.6–104.4) ± 6.8	92.2 (85.6–109.4) ± 9.7	100.9 (91.6–107.2) ± 6.2
Injury level-max angular velocity (rad/s)	Mean (range) ± SD	N/A	N/A	259 (257–261) ± 1.6	257 (253–262) ± 3.4
Recovery duration (minutes)	Mean (range) ± SD	8.3 (4.0–13.0) ± 4.5	13.3 (7.0–18.0) ± 5.7	36.2 (21.0–50.0) ± 10.9	14.8 (2.0–26.0) ± 10.7

To compare potential sex differences, all injured animals were examined at 24 h post-injury when extensive axonal pathologies are expected based on prior observation [14, 27, 28, 51]. In addition, this early time point is selected based on known sex differences in structural disruption of white matter at acute phases of clinical concussion [11] and its close relevance to our previous in vitro findings of sex differences in axonal structures/ outcomes [16]. The proposed animal number was anticipated to provide > 80% power and a Type I error of 0.05, especially considering the absence of axonal pathology in sham animals as demonstrated before [27, 28, 51]. All histologic experiments, analyses and quantification were performed blind to the injury status of the animal. All animal experiments were conducted in accordance with ARRIVE guidelines and protocols approved by the Institutional Animal Care and Use Committee at the University of Pennsylvania.

## Animal preparation, injury and recovery procedure

The animal preparation was carried out as previously described [9, 14, 27, 28, 51]. Animals were fasted for over 12 h before any surgical procedures and anesthesia was induced by intramuscular administration of midazolam (0.5 mg/kg) with dexmedetomidine (0.05 mg/kg) for injury procedure and midazolam with ketamine (20 mg/kg) for sacrifice procedure. Anesthesia was then maintained on a surgical plane via 2–5% isoflurane with snout mask and intubation. Animals’ statuses were monitored throughout the procedure.

Experimental concussion was performed using a HYGE pneumatic actuator, which can convert linear motion to angular (rotational) motion to produce impulsive head rotation of 110 degree in the coronal plane in 20 ms. In this model, the animal’s mouth was positioned with a padded bite plate and then the head was secured to the HYGE device with adjustable snout straps. This padded linkage assembly allowed precise control of swine head movement in the coronal plane. In this study, swine were subjected to precisely controlled coronal rotational injuries that ranged from 253 to 262 rad/s, with no difference between female and male injury group (Table 1). Rotational kinematics were recorded using angular velocity transducers (Applied Technology Associates) and calculated as previously noted [60].

Following injury, swine were immediately removed from the HYGE device, then extubated as prompted by either chewing on the endotracheal tube, swallowing or coughing, and continuously monitored in the housing unit for the duration of the recovery process. Preemptive analgesia of sustained release buprenorphine (0.2 mg/kg) was injected subcutaneously post-injury. Following previous description [60], recovery duration was defined as the time elapsed between extubation of animals and when animals were weight-bearing on all four limbs. Sham animals received all procedures described above except for the head rotation.

## Tissue preparation

Under terminal anesthesia, all animals were sacrificed at 24 h post-injury through transcardial perfusion with heparinized saline (2 L) followed by 10% neutral buffered formalin (8 L), as previously described [27, 28, 51]. The brain was then removed, extracted, and post-fixed in 10% formalin for 1 week. Subsequently, the brain was blocked at 5 mm in the coronal plane and tissue block was processed for either standard paraffin embedding in an automated tissue processor (Shandon Scientific Instruments) or standard TEM preparation (described in below section). Serial Sects. (8 µm) were cut on a Leitz rotary microtome (Leica) then mounted on Fisherbrand Superfrost/Plus microscope slides (Fisher Scientific).

The brain coronal level described in this study was designated according to the stereotaxic atlas of the pig brain such that in the antero-posterior direction, the anterior border of the posterior commissure was presented in the plane A 0.00 mm [17]. Then, the planes anterior to it were labeled as anterior (A) with the distance to this level. Here, continuous tissue blocks at plane A 5.50 mm, 10.50 mm, 15.50 mm, and 20.50 mm were used to systematically assess axonal pathologies (Supplementary Fig. 1a, online resource). These coronal levels include white matter that contains radiation of the corpus callosum (deep white matter) and subcallosal fasciculus, which were selected for analysis given their established biomechanical vulnerability to the injury [14, 27, 28, 51]. In addition, to test the hypothesis that female axons may be more selectively vulnerable than male axons related to axon size, tissue block at plane A 0.50 mm was selected to examine axon ultrastructure under TEM. Based on our extensive prior characterization, a consistently high number of swollen axonal profiles were observed at this level after injury [9, 27, 28].

## Immunohistochemical (IHC) staining and quantification

Standard IHC techniques were performed as previously described [27, 28, 49, 51, 52]. Swine brain tissue sections were deparaffinized with xylene and rehydrated using a series of graded ethanol (100% and 95%) and water. Sections were then incubated with 3% hydrogen peroxide (Carolina Biological) for 15 min to quench endogenous peroxidase activity. Antigen retrieval was followed by heating in Tris EDTA buffer (pH 8.0) using TintoRetriever Pressure Cooker (Bio SB). Sections were then blocked with normal horse serum (Vector Labs) in Optimax buffer (BioGenex) and incubated with primary antibody of amyloid precursor protein (APP) (Millipore, MAB348, 1:60,000) or myelin basic protein (MBP) (Cell Signaling Technology, 78896, 1:2000) overnight at 4 °C. Next day, biotinylated secondary antibody (Vector Labs) was applied for 30 min followed by avidin biotin complex (Vector Labs) incubation for another 30 min. Visualization was done by DAB peroxidase substrate kit (Vector Labs) and counterstaining with hematoxylin (Surgipath, Leica Biosystems). Sections were scanned at 20 × with an Aperio ScanScope and annotation of each APP-immunoreactive profile was achieved using Aperio ImageScope software (Leica Biosystems).

Quantification of APP-immunoreactive axonal profile was followed by previous studies that have extensively characterized the pattern and distribution of APP axonal pathology in this swine model of concussion [9, 14, 27, 28, 51]. Specifically, an individual APP-immunoreactive axonal profile was defined as a swollen bulb, morphologically injured, or varicose profile and counted once for analysis. First, we mapped individual APP-immunoreactive profile in whole brain section of both injured female swine and males. Given the known biomechanical vulnerability of this model, we further performed focused examination and quantitative analysis of white matter regions that contain left radiation of the corpus callosum (deep white matter) and subcallosal fasciculus adjacent to the left lateral ventricle, where extensive axonal pathologies were consistently shown for both sexes (Supplementary Fig. 1b, online resource). We provided an estimation of the No. of APP-immunoreactive profiles spanning four continuous coronal levels of the brain. For each of the coronal levels, approximately 4 mm^2^ area at the center of left deep white matter and 1 mm^2^ area at subcallosal fasciculus were systematically surveyed for positive APP-immunoreactive axonal profiles (Supplementary Fig. 1b, online resource, inset white box). The No. of APP-immunoreactive profiles was counted for whole section and in a given field, converted to per unit area (mm^2^), and averaged for individual animal for statistical analysis. Quantification was performed independently by two investigators, blind to the injury status, with results showing excellent inter-rater reliability (interclass correlation coefficient (ICC) of 0.94).

## Immunofluorescent (IF) staining, confocal imaging, and quantification

IF staining was carried out similarly to the IHC procedures, except for the hydrogen peroxide quenching step was omitted as previously described [27, 28, 49, 51]. Sections were then incubated overnight at 4 °C with primary antibodies of Nav1.6 (Alomone Labs, ASC-009, 1:200), Caspr (from Stephen Waxman, Yale University, generated against the GST fusion protein composed of the entire cytoplasmic domain of rat p190/Caspr (contactin-associated protein) (GST–190CT), 1:2000) [42], and APP (Millipore, MAB348, 1:1000). The next day, secondary antibodies conjugated with Alexa Fluor 488/568/647 (ThermoFisher, 1:250) were applied on sections for 60 min. VECTASHIELD Vibrance antifade mounting medium (Vector Labs) was used for coverslipping.

For visualization, sections were imaged using a Zeiss LSM 880 Airyscan confocal microscope with a 63 × oil immersion objective (1.40 NA) and 488, 561, and 633 laser lines for each fluorophore as previously described [51]. The Airyscan confocal used a multichannel area detector with 32 elements to collect all the light from an Airy disk simultaneously with no need to set the pinhole size. Images were acquired with z-stack (numerical zoom of 2, a total of 10 steps with each z-step of 0.22 μm) and prepared using ImageJ. Surface rendering was performed for 3D reconstruction using Imaris software (GraphPad, Bitplane, v.9.7.2).

Quantitative analysis of Nav1.6 was conducted independently by two investigators, blind to the injury status, again showing excellent inter-rater reliability (ICC of 0.97)). Following previous description [51], for individual animal, two non-overlapping regions of interests (ROIs) from left deep white matter and one ROI from subcallosal fasciculus were surveyed in each of the four coronal levels (approximately covering a total area of 0.12 mm^2^ for one level and 0.48 mm^2^ per animal). These ROIs were selected in close proximity to existing APP-immunoreactive axonal profiles. The loss of Nav1.6 was quantified by counting the numbers of Nav1.6 void nodes and then calculated as a percentage by dividing the numbers of void nodes by the numbers of total NOR observed. Caspr staining labels septate-like paranodal space surrounding the NOR and was used as a reference to characterize the void node morphologic phenotype (paired Caspr domain in the absence of Nav1.6). In addition, the fluorescent intensities of Nav1.6 were measured using ImageJ and normalized against background fluorescent signals to generate relative expression [Integrated Density—(Area of selected area * Mean fluorescence of background readings)]. Since the presence of Nav1.6 is diffuse and its loss after experimental concussion is widespread across the brain white matter [51], measurements of Nav1.6 were then averaged for each animal for statistical comparison.

## Transmission electron microscopy (TEM) procedure and quantification

Brain tissue at the coronal level of plane A 0.50 mm was used for TEM preparation, processing, and analysis. The tissue block was post-fixed with a mixture of 2.5% glutaraldehyde and 2% paraformaldehyde (Electron Microscopy Sciences) in 0.1 M sodium cacodylate buffer (pH 7.4). Samples cut from the corpus callosum were selected for cross-sectional examination of white matter axons. After subsequent washes with 0.1 M sodium cacodylate buffer, tissues were post-fixed in 2% osmium tetroxide for 1 h at room temperature and rinsed in diH_2_O prior to *en bloc* staining with 2% uranyl acetate. After dehydration through a graded ethanol series, samples were infiltrated and embedded in EMbed-812 (Electron Microscopy Sciences). Thin Sects. (75 nm) were then stained with uranyl acetate and lead citrate.

Ultrastructural images were acquired using a JEOL JEM 1010 electron microscope fitted with a Hamamatsu digital camera and AMT Advantage image capture software at 10,000 × magnification for quantitative analysis. A higher magnification at 25,000 × was used for figure illustration. For analysis, an approximately 6000 μm^2^ area selected in a systematic random fashion was surveyed for individual injury-F and injury-M group animals, and an 11,000 μm^2^ area was surveyed for each sham-F and sham-M group. While there may be some artifact in myelin lamination possibly owing to fixation, normal myelinated axons were selected for measuring axon area, g-ratio, and frequency of fibers based on their intact axolemma and no presence of apparent loss of axon content, including neurofilament and normal mitochondria as previously described [16, 30, 50]. In contrast, injured axons were distinguished based on the presence of empty of axon cytoplasm/ neurofilament or apparent formation of vacuoles/ degenerated mitochondria/ lysosomes in the appearance of a swollen axon. Note that when measuring axon area in injured animals, only axons without the morphologic changes described above were included in analysis to capture caliber changes after injury.

Axon area was measured using ImageJ (NIH, USA). Axon diameter was then converted by 2 $\times \sqrt{area\div \pi }$ and g-ratio was calculated as $\sqrt{inner \, myelin \, area\div outer\, myelin\, area}$. The percentage of injured axons was quantified as numbers of injured axons/ numbers of total axons surveyed. For statistical analysis, the averaged measurements from individual animals were used to compare differences between groups. All TEM quantitative analyses were performed independently by multiple investigators, blind to the injury status, with results demonstrating excellent inter-rater reliability (ICC of 0.95).

## Statistical analysis

Statistical analysis was performed using GraphPad Prism statistical software (GraphPad Software Inc.). Data were presented as scatter plots or bar graphs and expressed as mean ± standard error of mean. A two-sample *t* test was used to determine differences between two groups [28, 51]. Linear regression was used to correlate recovery duration and the extent of axonal pathologies. The frequency of axon fibers in TEM studies was depicted by Gaussian distribution and compared using Kolmogorov–Smirnov test. A *p* value less than 0.05 was considered significant for all analyses, with * indicating *p* < 0.05, ** for *p* < 0.01, *** for *p* < 0.001, and **** for *p* < 0.0001. The inter-rater reliability was assessed by ICC. The ICC values were interpreted as follows: < 0.40, poor; 0.4–0.6, fair; 0.6–0.75, good; and 0.75–1.0, excellent [12].

## Results

### Animal characteristics, injury kinematics, and recovery duration following experimental concussion

Both female and male swine were selected at a similar range of age (6 to 7 months) (Table 1). While the female swine averaged slightly lower body weight than the males, there was no difference in brain tissue mass (Supplementary Fig. 2a, b, online resource). Closely matched brain mass between animals was important for this study, since larger brains are predicted to undergo greater tissue deformation and axonal pathology than smaller brains under the same parameters of head rotational acceleration [22, 35]. Here, setting the same head rotation parameters for each injury, the maximum angular velocities were found to be the same between sexes (Table 1 and Supplementary Fig. 2c, online resource). In addition, there was no significant difference between sexes in additional kinematic parameters, including injury velocity magnitude, acceleration peaks, and acceleration duration, as reflected by the captured velocity and acceleration traces (Supplementary Fig. 2d–i, online resource). Immediately following injury, female swine sustained a significantly longer recovery duration than males, which was also greater than sham animals (Table 1 and Supplementary Fig. 3a, online resource).

## Experimental concussion results in greater number of swollen axonal profiles in females, identified by APP immunohistochemistry

Historically, the ‘gold standard’ histopathological evidence of DAI has been the identification of APP-immunoreactive swollen axonal profiles in a multifocal pattern across the white matter [1, 6, 20]. These swellings form as a result of impaired axonal transport arising from mechanical disruption of the axon cytoskeleton [20]. Here, we quantified the numbers (No.) of APP-immunoreactive axonal profile following previous description [27, 51], defined as a swollen axonal bulb or individual morphologically injured or varicose profile, particularly in brain white matter where established biomechanical vulnerability was shown in this model [9, 14, 27, 28, 51].

**Fig. 1:**
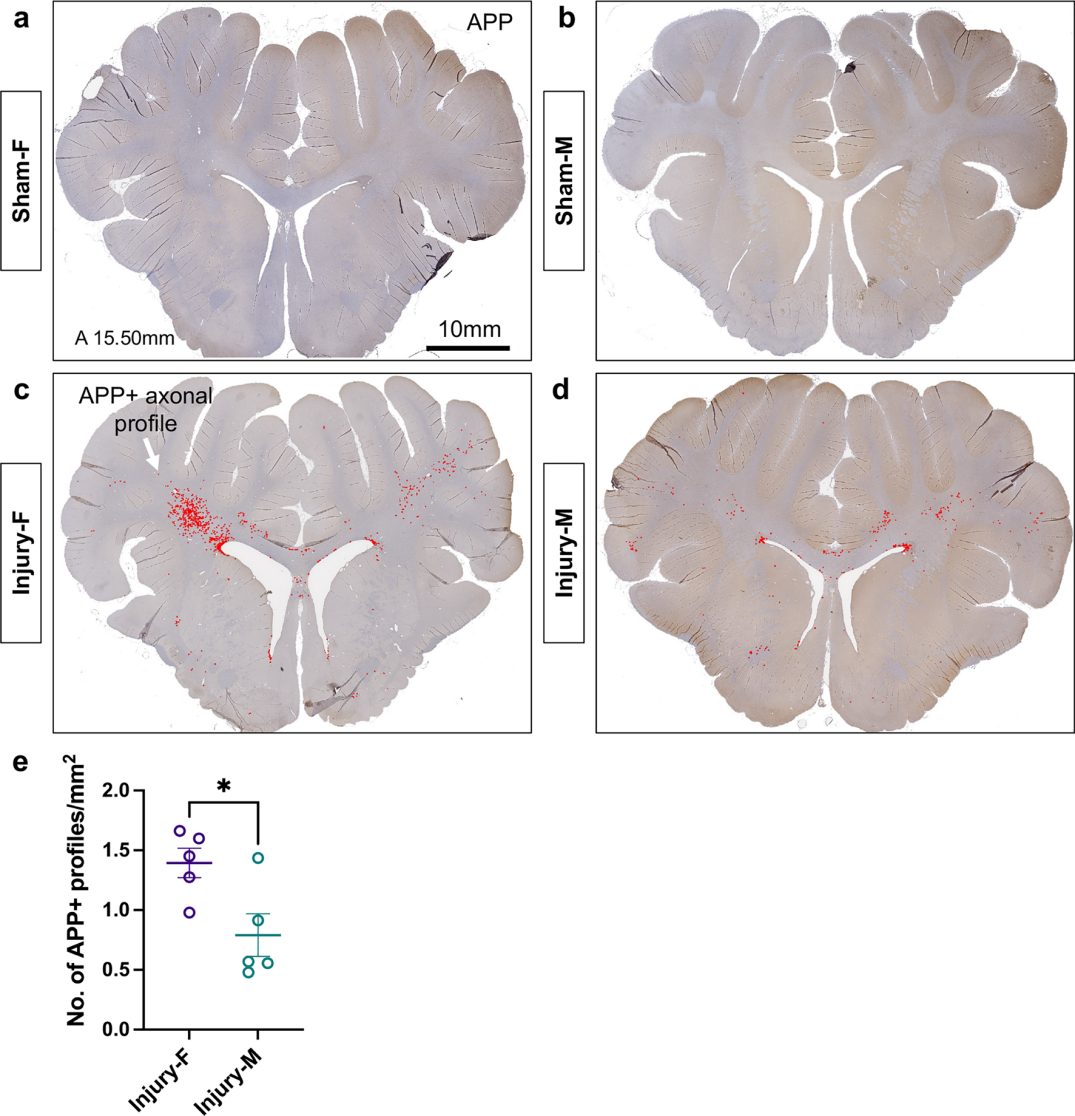
Extent of APP axonal pathology following experimental concussion. Representative whole brain section map of annotated APP-immunoreactive profiles (at A 15.50 mm level) in sham female (**a**) and male (**b**) swine, as well as injured female (**c**) and male swine (**d**). Red-dot labels annotate individual APP-immunoreactive axonal profiles. Scale bar = 10 mm. **e** Significantly greater numbers of APP-immunoreactive axonal profiles per whole brain section were observed in injured females than injured males at 24 h post-injury. Each dot represents individual animal’s mean

**Fig. 2:**
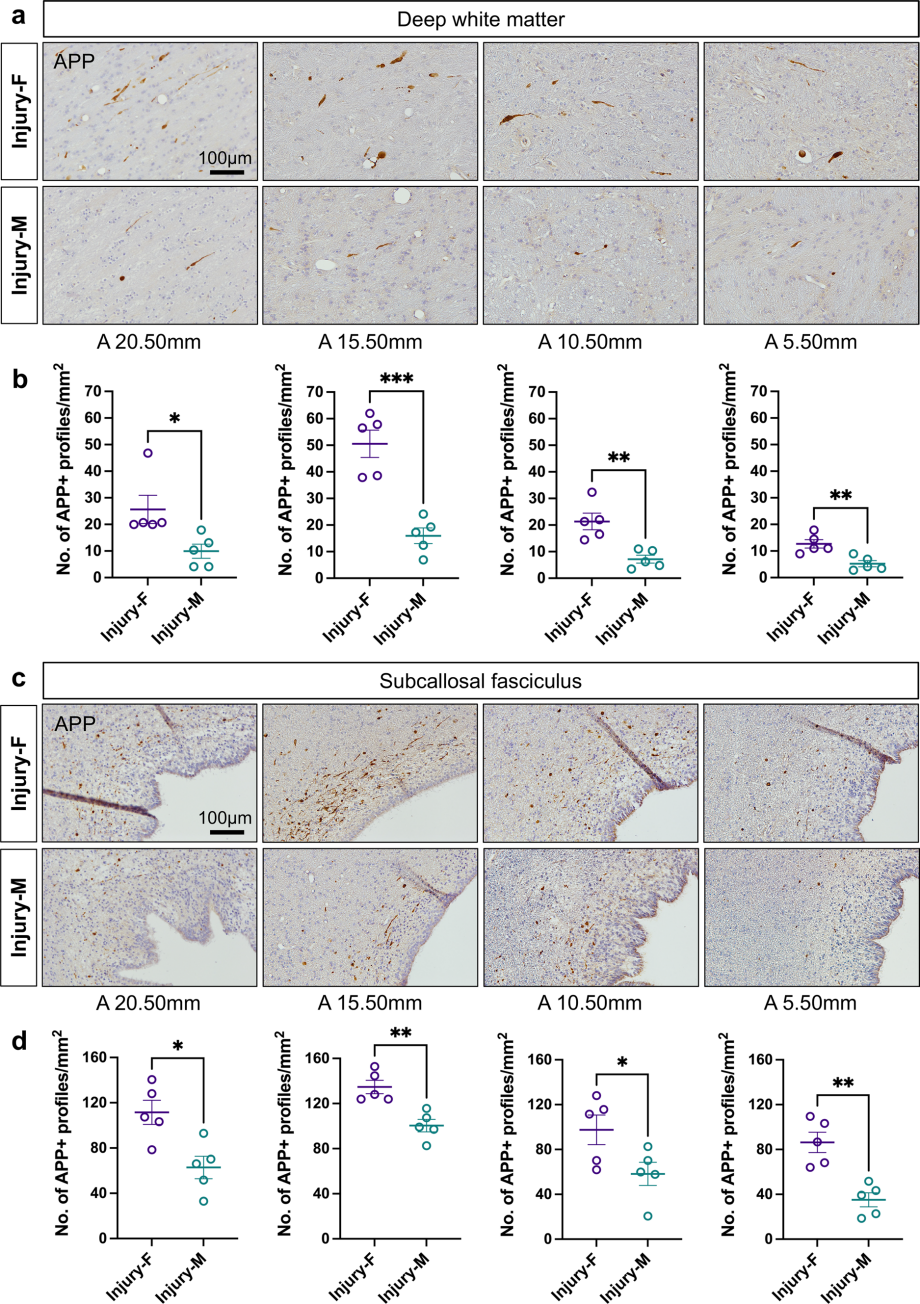
Greater numbers of APP swollen axonal profiles in females after injury. **a**, **b** Through focused examination across four continuous brain coronal levels, significantly more numbers of APP-immunoreactive axonal profiles per unit area (mm^2^) at deep white matter were found in injured female swine than injured males at 24 h post-injury. Various microscopic swollen axonal profiles were displayed, including characteristic periodic varicosities/ beading, fusiform profiles and terminal bulbs, which indicate axonal transport interruption. Scale bar = 100 μm. **c**, **d** Similarly, significantly greater numbers of APP-immunoreactive axonal profiles at subcallosal fasciculus were found in injured female swine than injured males

No APP-immunoreactive profiles were identified in the sham animals in either sex (Fig. 1a, b and Supplementary Fig. 4, online resource). In contrast, whole-section map of APP-immunoreactive profiles showed axonal pathology in both injured female swine and males, with a similar distribution relevant to the dynamic mechanical deformation of brain tissue but differing in extent (Fig. 1c, d). Notably, at 24 h post-injury, a greater extent of APP-immunoreactive axonal profiles were identified in female swine compared to males throughout whole section (Fig. 1e), and particularly at white matter localized to the left radiation of the corpus callosum (deep white matter) and subcallosal fasciculus adjacent to the lateral ventricle (Fig. 2). This difference was consistent throughout continuous coronal levels of the brain. The extent of APP axonal pathology was positively correlated with injury recovery duration (Supplementary Fig. 3b and Supplementary Table 1, online resource). As in previous studies using this model, these swollen axonal profiles were morphologically similar to those found in human TBI [6, 20, 27, 51] and presented as characteristic periodic varicosities or beading along the axon, fusiform profiles, and terminal bulbs (Fig. 2a, c). In addition, overt loss of myelination (MBP) was not detected in the tissue (Supplementary Fig. 5, online resource).

## Females display more widespread loss of axonal NaChs after concussion

Previous studies using an in vitro model of traumatic axonal injury [24, 62], the current swine model of concussion, and human moderate or severe TBI cases [51] demonstrated that widespread and progressive disruption of axonal NaChs represents another important phenotype of DAI. Here, potential sex differences in NaCh integrity were investigated, with a specific focus on the Nav1.6 that is normally positioned at the NOR in myelinated axons.

**Fig. 3:**
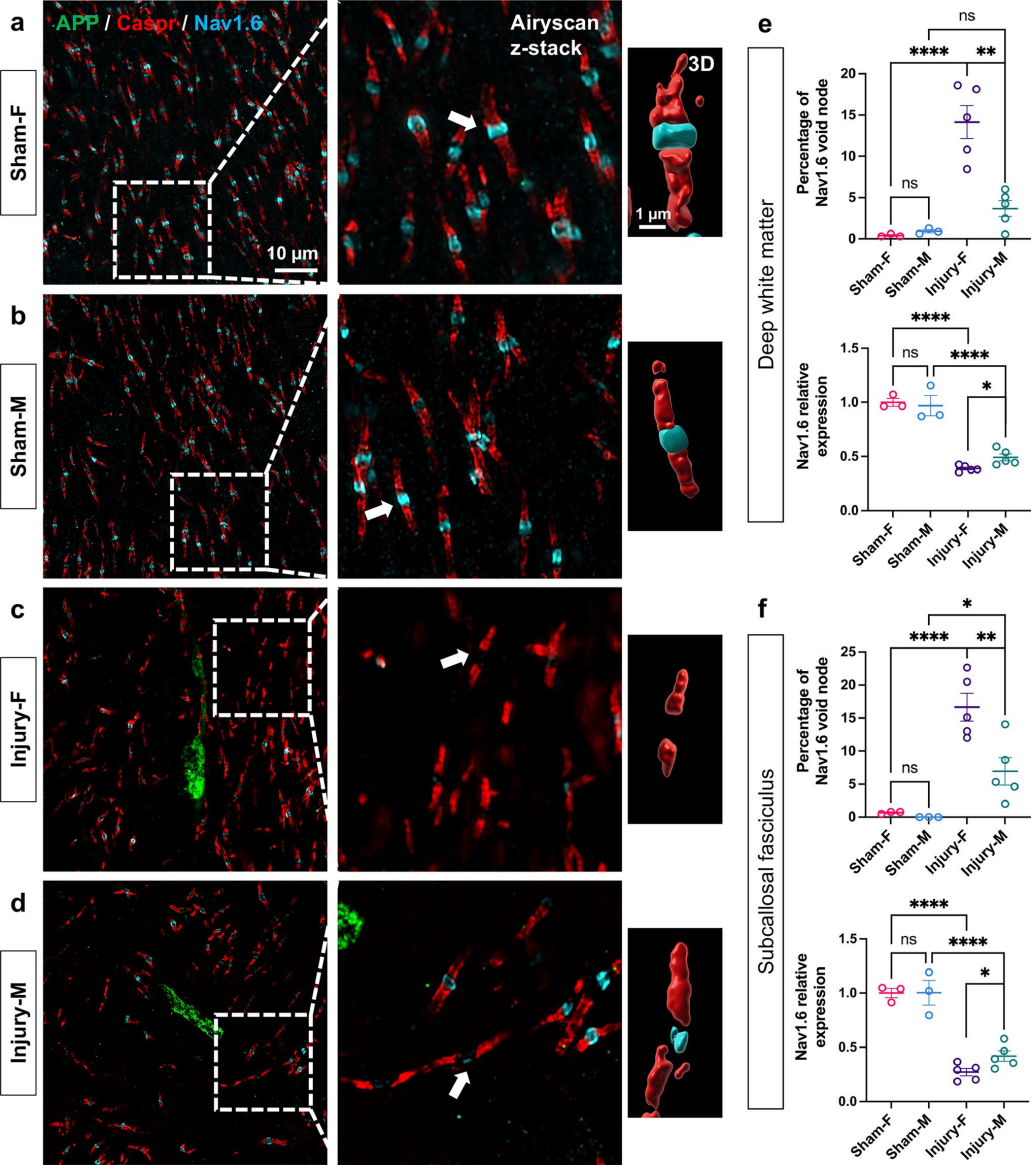
Widespread loss of Nav1.6 following experimental concussion. **a**, **b** Representative images from brain deep white matter show normal Nav1.6 expression (cyan) in both sham female and male animals within the NOR along with double Caspr (red) labeling the paranodal space. **c**, **d** After experimental concussion, injured female animals sustained greater loss of Nav1.6 in white matter. White arrow labels the characteristic Nav1.6 loss from the enlarged view, which is further confirmed by 3D reconstruction. **e**, **f** The relative expression of Nav1.6 was similar between sham female and sham male animals, yet extensive losses of Nav1.6 were identified in both injured female and injured male animals at both deep white matter and subcallosal fasciculus. In contrast to injured male swine, injured females showed significantly more numbers of Nav1.6 void node and significantly less expression of Nav1.6. Each dot represents individual animal’s mean

Based on prior characterization [51], we quantified the relative expression of Nav1.6 immunoreactivity along axons of normal diameter in the same white matter regions where extensive APP axonal pathology was identified. Normal Nav1.6 immunoreactivity was expressed at NOR in axons and surrounded by paranodal Caspr, in both sham female and sham male in all microscopic fields of white matter, with no observable loss or difference in the relative density of Nav1.6 between the sexes, based on characteristic fluorescent signatures (Fig. 3a, b, e, f, and Supplementary Fig. 6a, b, online resource). At 24 h post-injury, widespread loss of Nav1.6 expression was identified along axons that were often nearby but distinct from APP-immunoreactive axonal profiles (Fig. 3c, d and Supplementary Fig. 6c, d, online resource), with injured female axons displayed a greater number of Nav1.6 void nodes (paired Caspr domain in the absence of Nav1.6 at the NOR) and relatively fewer Nav1.6 fluorescent-labeled axons than injured male axons at both deep white matter and subcallosal fasciculus (Fig. 3e, f). These morphologic changes were confirmed by the corresponding 3D surface reconstruction showing reduced nodal Nav1.6 expression. Similar to APP axonal pathology, the extent of Nav1.6 loss was positively correlated with injury recovery duration (Supplementary Fig. 3c and Supplementary Table 1, online resource).

## Sex differences in axon ultrastructure

Since multiple axonal pathology phenotypes were more widely observed in female brain white matter, we hypothesize that female axons may be more selectively vulnerable than male axons when subjected to the same biomechanical loading of concussion, potentially related to axon size. We first examined sex differences in normal axonal architecture at nanoscale and then explored how structural differences could affect the formation of axonal pathologies.

**Fig. 4:**
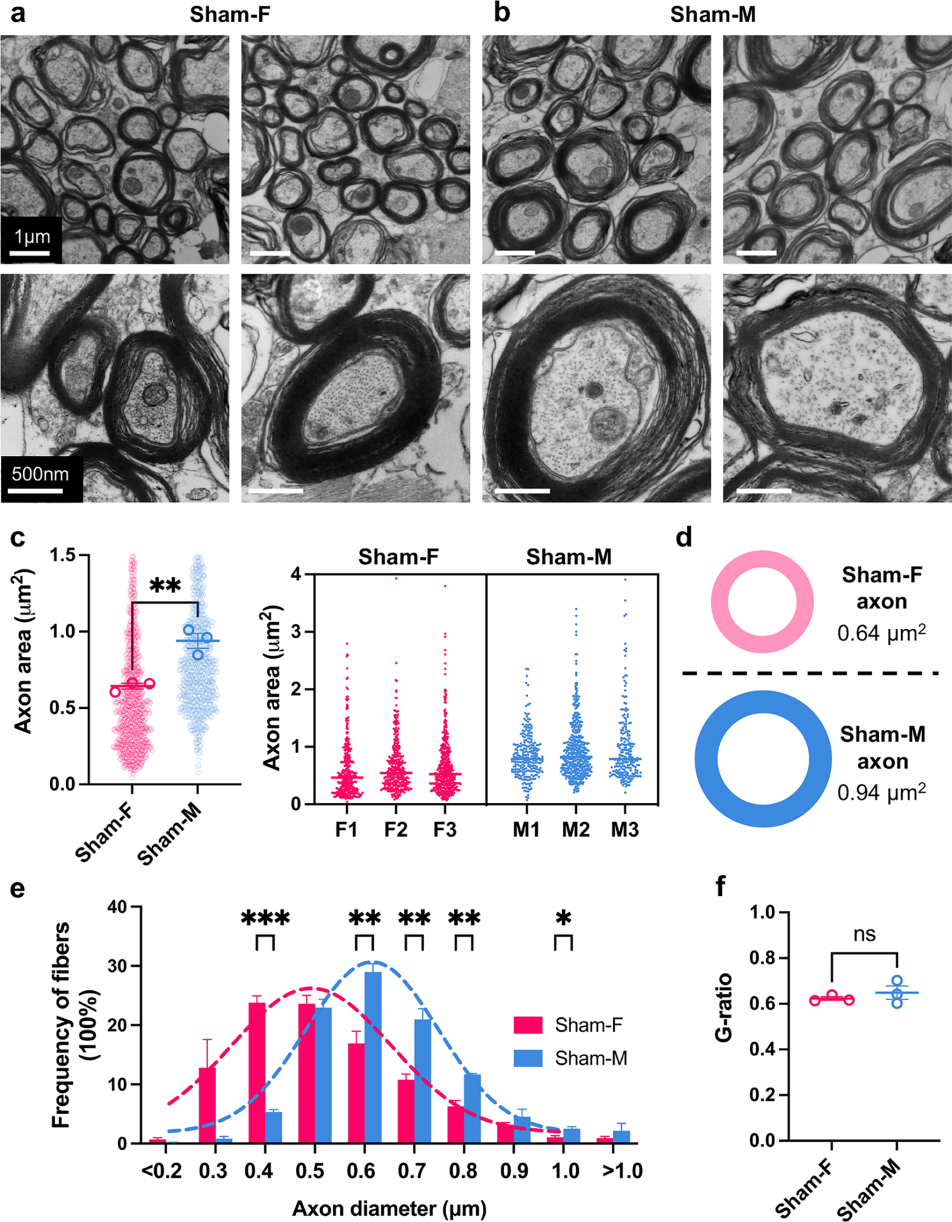
Axon caliber differences between sham female and male swine brains. **a**, **b** Cross-sectional view of axons captured by electron micrographs of corpus callosum tissue from sham female and sham male animals. The lower panels show representative enlarged view of single myelinated axons. **c** On average, female axons were significantly smaller than male axons. The solid dot represents individual animal’s mean, while the shadowed dot represents measurement from a single axon. Data from each animal were visualized in the right panel, showing consistent distribution of axon sizes from both sexes. **d** Illustration depicts sex difference in axon areas between sham female and sham male animals. **e** Histogram of axon fiber frequency shows differences in axon diameter (μm) distribution between female and male axons (best-fitting curve in dashed line with Gaussian distribution). **f** No difference was observed in g-ratio

In sham animals, cross-sectional TEM examination of myelinated axons at the corpus callosum revealed that, on average, female swine axon area was approximately 32% smaller compared to male axons (0.64 μm^2^ vs. 0.94 μm^2^) (Fig. 4a–d). Histogram representation of axon fiber frequency further demonstrated that the percentage of small caliber axons (< 0.5 μm) were significantly higher in females than in males (Fig. 4e). This is supported by cumulative frequency analysis (Supplementary Fig. 7a, online resource). In contrast, the presence of relatively large caliber axons (> 0.5 μm) was more profound in males than in females. In addition, TEM showed that both female and male axons displayed a similar degree of MBP with no difference in g-ratio (Fig. 4f).

## Smaller axons are more selectively damaged after concussion

**Fig. 5:**
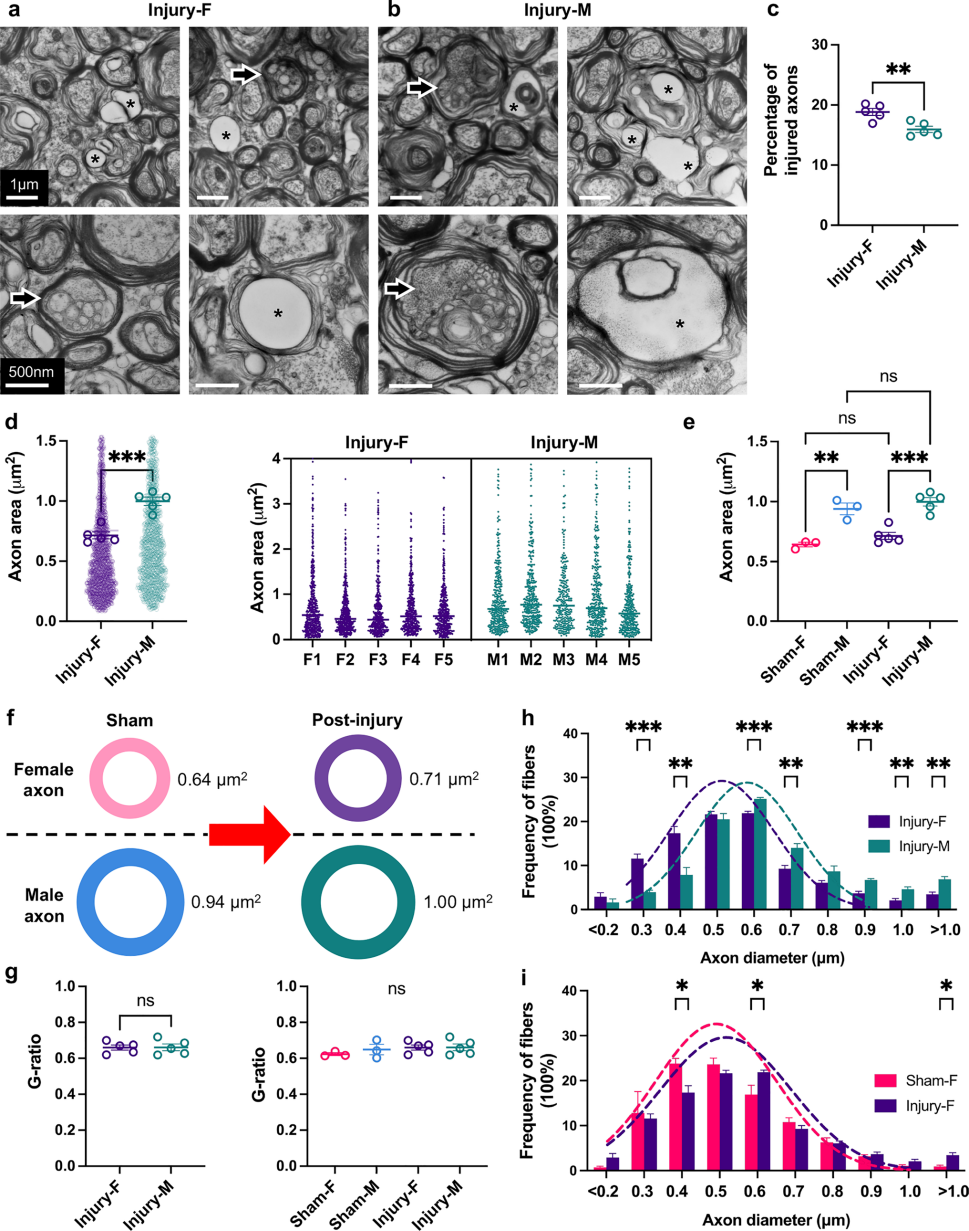
Changes in axon caliber and ultrastructures following experimental concussion. **a**, **b** Cross-sectional view of white matter axons after injury. Various ultrastructural changes were observed in both injured female and male axons, including accumulation of degenerated mitochondria/ lysosomes in the appearance of swollen axons (arrow) and vacuole formation/ clearance of axon cytoplasm (asterisk). **c** Percentage of injured axons was significantly higher in injured female than male animals, consistent with the microscopic axonal pathology observation. **d**, **e** After injury, female axons were still significantly smaller in size than male axons. Nonetheless, averaged axon area was similar between injured and sham animals in either sex. **f** Illustration depicts axon area difference between injured female and injured male animals, as well as increases in axon areas after injury within each sex. **g** No obvious difference was noted in g-ratio. **h**, **i** Axon fiber frequency histogram shows differences in distribution between male and female animals, as well as shifts in distribution between the female axons after injury and sham female axons

Experimental concussion disrupted axon ultrastructure as indicated by clearance of axon cytoplasm, vacuole formation and abnormal accumulation of degenerated mitochondria or lysosomes, which appears to identify a swollen axonal profile (Fig. 5a, b). Consistent with IHC examination, the percentage of damaged female axons under TEM were much higher than those in male (Fig. 5c), supporting a greater extent of axonal damage down to the nanoscale level in female brain white matter.

At 24 h post-injury, the average female axon areas remained significantly smaller (29%) compared to males (0.71 μm^2^ vs. 1.00 μm^2^) (Fig. 5d–f). Again, axon fiber distribution analysis showed that the presence of small axons (< 0.5 μm) was more frequent in females, whereas large caliber axons (> 0.5 μm) were more predominant in males (Fig. 5h and Supplementary Fig. 7, online resource). Notably, we identified that, within each sex, the average axon area was relatively increased after injury (11% increase in female and 6% in male), indicating a selective loss of small caliber fibers for both sexes, but to a greater extent in females (Fig. 5e, f). Specifically, the percentage of small caliber axons (0.3–0.4 μm) in injured females was significantly reduced comparing to that of sham-injured sham female control. In addition, the percentage of large female axons with a diameter between 0.5–0.6 μm, as well as over 1 μm, were significantly increased after injury (Fig. 5i). This is again supported by cumulative frequency analysis (Supplementary Fig. 7a, online resource). Finally, there was no apparent change in g-ratio after injury and no difference between sexes (Fig. 5g).

These observations demonstrate that small caliber axons are more selectively vulnerable to be damaged and undergo degeneration than larger caliber axons under similar biomechanical loading conditions. Considering a generally smaller axon size in females, our data suggest that this plays a key role in the greater extent of axonal cytoskeletal damage and NaCh disruption following concussion.

## Discussion

For experimental concussion in swine performed with the same biomechanical loading conditions of head rotational acceleration, female brains displayed a greater extent of APP accumulation in swollen axonal profiles and more widespread loss of Nav1.6 along injured axons than observed for males. The more extensive axonal pathology in females also appeared associated with a longer injury recovery duration compared to males. We also discovered that axon damage leading to degeneration acutely after concussion was related to axonal diameter since there were substantially fewer small caliber fibers after injury for both sexes. However, we also found that female axons were on average smaller than male axons and that female swine had a proportionately greater loss of small caliber fibers after concussion. Accordingly, these findings indicate that sexual dimorphism in the average size of axons predisposed females to greater loss and dysfunction of axons after concussion. These sex differences in average axon architecture and selective loss of small caliber fibers after concussion suggest a potential mechanistic basis underlying previously observed sex differences in concussion outcomes in humans.

Our present data identify the first evidence that white matter in the female brain exhibits a greater extent of axonal pathology phenotypes than males under the identical head rotational acceleration forces of concussion. Specifically, brain tissue deformation during head rotational acceleration appears to cause greater disruption of axonal transport in female axons than male axons. This induces protein accumulation in swellings, which leads to secondary disconnection and degeneration. The various morphologies of swollen axonal profiles observed in the present study included characteristic varicose axonal swellings along injured axons and terminal bulbs at the ends of disconnected axons in both sexes, which were identical to those identified in human TBI [6, 20, 27, 51]. While there is evidence that some swollen axons can recover to normal size and function after injury [40], the majority of these swollen axonal profiles are thought to go on to secondary axonal disconnection and degeneration [57].

Consistent with our previous characterization [9, 27, 28, 51], the distribution of APP-immunoreactive swollen axonal profiles was multifocal and spatially localized to white matter tracks that have been predicted to undergo greater mechanical deformation during head rotational acceleration, based on computational models [29, 64]. The current data indicate that the most distinct sex difference in APP immunoreactive axonal pathology occurs at the radiation of the corpus callosum. This is consistent with neuroimaging studies of human sports-related concussion, where females had reduced-fractional anisotropy (FA) in a similar corona radiata region than males [11, 48]. Notably, in human, these commissural and long association white matter fibers (i.e., subcallosal fasciculus or known as superior occipitofrontal fasciculus) are thought to connect and relay information between multiple cortical representations that are important for visual, motor, and executive functioning. Selective axonal damage in these tracks due to concussion may be therefore implicated in clinical outcomes, such as oculomotor dysfunction, difficulty concentrating, and slowed processing speed, altogether potentially contributing to the greater susceptibility of females to post-concussion symptoms and prolonged recovery [15, 48].

Based on previous in vitro data, we hypothesized that the in vivo sex difference in axonal pathology may be attributed to axon size in cross-sectional caliber. In the present study, using TEM analysis of examining the swine corpus callosum, we found that female axons are on average 32% smaller than male axons. This TEM observation agrees with our previous in vitro finding [16], as well as other microscopy and MRI studies on human and non-human primates [38, 39, 55]. While it is unknown why this sex differences evolved, it has been suggested that the number of axons for a given white matter tract may be roughly the same for females and males, with the greater axon size for males accounting for larger white matter volumes [31, 41].

Here, we also provide the first evidence that small caliber axons are more susceptible to degeneration than large axons from the same white matter tracks, in both female and male swine after injury. Specifically, for both sexes, TEM analysis demonstrated that the average size of axons increased after injury suggesting that there was selective degeneration of small caliber fibers. Moreover, we found that there is a greater proportion of small caliber axons in female white matter compared to males. Accordingly, this appears to predispose female axons to a greater extent of axonal transport interruption and resulting swelling, which can lead to disconnection and degeneration.

The selective vulnerability of small caliber axons to become injured may result from greater damage to their microtubules (MTs). Axonal MTs are aligned in a parallel orientation and are cross-linked with adjacent MTs via the predominant MT stabilizing protein, tau. During normal daily activities, axons can tolerate substantial quasistatic stretching and return to their resting lengths unharmed [3]. Computational modeling has shown that during quasistatic stretch, adjacent axonal MTs slide past each other, causing the cross-linking tau proteins to unfurl from their resting conformation via sequential release of H-bonds as they are extended [3]. However, under dynamic stretching of axons during concussion, the forces pulling against many H-bonds of extending tau proteins all at once causes dynamic high stress at the respective tau-MT binding sites. This induces immediate rupture of axonal MTs, as predicted by computational models and seen by TEM after in vitro axonal stretch injury [2, 3, 54]. The rupture points of broken MTs impedes relaxation of the axon to the original length, resulting in the formation of undulations along injured axons that eventually become sites of swelling due to transport interruption, as seen in in vitro axon stretch injury, swine concussion, and human TBI [2, 16, 27, 45, 51, 54]. The relatively sparser array of MTs in small caliber fibers compared to large caliber fibers appears to place their respective MTs at particular risk of mechanical breaking during dynamic stretch, as demonstrated by computational modeling [16].

Although axonal swellings are historically considered as the hallmark feature of DAI, we have found that there are multiple phenotypes of axonal pathology that fall under the spectrum of DAI [27, 51]. Previously, using an in vitro model of dynamic axonal stretch injury, we observed immediate non-inactivation of axonal NaChs, leading to massive sodium influx [62]. This was found to trigger a pathologic influx of calcium into injured axons, activation of calpain, and the resulting proteolytic degradation of NaChs [24, 56]. This mechanism was recently corroborated in vivo in this swine concussion model and in human TBI, where an expansive loss of NaChs was observed in addition to loss of and/or mislocation of associated nodal proteins [51]. Notably, axons displaying NaCh loss do not appear to have swellings, yet can be found nearby APP-immunoreactive swollen axonal profiles in the same tract. In the present study, we found that females displayed a greater loss of axonal Nav1.6 throughout the white matter after concussion compared to males. In comparison to APP-immunoreactive axonal swellings, this NaCh loss affected far more axons per tract in expansive white matter, appearing in regions with no axonal swellings. The loss of axonal NaChs reflects a loss of ionic homeostasis that can disrupt the central component of axonal signaling machinery, the generation of action potentials [24, 62]. Thus, in addition to physical loss of brain-network connectivity through axon degeneration as a consequence of axonal swelling, the loss of axonal NaChs in concussion may represent a potential physiologic disruption of functional connectivity and potentially account for the longer recovery duration in females. Together, axon degeneration and dysfunction provide plausible substrates that may contribute to concussion symptoms.

While our current findings identify sex differences in the extent of axonal pathologies in the acute setting following concussion, we have previously observed ongoing axonal degeneration for months and even years following TBI in both humans and swine [10, 26, 45]. Further, there is increasing recognition that TBI is a major modifiable risk factor for neurodegeneration, including Alzheimer’s disease [43] and chronic traumatic encephalopathy [46], Here, progressive axonopathy is thought to be a key driver of TBI-related neurodegeneration since potential protein substrates for tau and amyloid pathologies may have their start in the milieu of axonal injury [25, 46]. Accordingly, it may be important to explore if sex differences in the extent of these progressive changes persist and to examine potential links between progressive axonal pathology in human concussion with the onset of TBI-related neurodegeneration and the risk of dementia. In addition, while recognizing that axon degeneration is associated with demyelination to some extent, such as in multiple sclerosis, overt changes in MBP were not detected in this concussion model. This observation is consistent with our previous characterization [10, 61], and perhaps warrants a closer examination of specific myelin breakdown product or degradation in future [23].

In addition to our current findings of sex differences in the extent of axonal pathologies after concussion, other factors that could affect different clinical outcomes have been proposed. For example, sex differences in neck strength have been suggested to influence the extent of head rotational acceleration given similar head impacts, with greater accelerations occurring to the female brain [59]. Alternatively, sex hormone difference has also been proposed to affect sex specific effects of concussion. However, despite the correlation between higher levels of progesterone and estrogen and worse concussion symptoms [65], contradictory clinical data show improved outcomes following head injury in patients taking progesterone [63], thus making its role less clear.

We recognize several limitations of this study. For example, while this study represents a first step to identify sex differences in acute axonal pathologies in focused white matter regions known to be selectively vulnerable to develop APP swollen axonal profiles along with widespread NaCh losses in swine model of concussion, a more comprehensive spatial distribution of axonal pathologies needs to be established. In addition, although it is inferred that sex difference in NaCh loss may be an important molecular substrate for brain network disruption that contributes to concussion symptoms due to the loss of action potential machinery, electrophysiological studies are required to examine the decrease in NaChs in relevance to functional outcomes. Lastly, the use of TEM to determine axon size does not allow direct comparisons with IHC identification APP + swellings or NaCh loss injured axons, which may be further characterized using immuno-EM techniques.

Together, these data identify sex differences in the extent of axonal pathologies in brain white matter acutely following experimental concussion, which appears related to sex differences in axon diameter. Notably, these findings provide a plausible pathologic substrate that may contribute to the sex differences in clinical outcomes of concussion. In addition, the current data support the examination of non-invasive approaches to identify potential sex differences after concussion, including blood biomarkers of axonal pathology and advanced neuroimaging, to identify the relative changes in white matter volume and connectivity.

### Supplementary Information

Below is the link to the electronic supplementary material.

Supplementary file1 (PDF 7767 kb)

## Data Availability

All data are available in the main text and the supplemental materials.
