# Supplementary material for: Sex differences in the extent of acute axonal pathologies after experimental concussion
Source: Acta Neuropathol. 2024 May 5;147(1):79. doi: 10.1007/s00401-024-02735-9 (PMC11070329; doi:10.1007/s00401-024-02735-9)
Supplement: Supplementary file 1 — Supplementary file1 (PDF 7767 kb) [file 401_2024_2735_MOESM1_ESM.pdf]

Supplemental Information

Sex differences in the extent of acute axonal pathologies after experimental concussion

Authors: Hailong Song[1], Alexandra Tomasevich[1], Andrew Paolini[1], Kevin D. Browne[1,2], Kathryn L. Wofford[1,2], Brian Kelley[1], Eashwar Kantemneni[1], Justin Kennedy[1], Yue Qiu[1], Andrea L.C. Schneider[4,5], Jean-Pierre Dolle[1], D. Kacy Cullen[1,2,3], Douglas H. Smith[1,*].

Affiliations

[1]Department of Neurosurgery, Center for Brain Injury and Repair, University of Pennsylvania, Philadelphia, PA 19104, USA

[2]Center for Neurotrauma, Neurodegeneration & Restoration, Michael J. Crescenz Veterans Affairs Medical Center, Philadelphia, PA 19104, USA

[3]Department of Bioengineering, School of Engineering and Applied Science, University of Pennsylvania, Philadelphia, PA 19104, USA

[4]Department of Neurology, University of Pennsylvania, Philadelphia, PA 19104, USA

[5]Department of Epidemiology, Biostatistics, and Informatics, University of Pennsylvania, Philadelphia, PA 19104, USA

***Corresponding Author**

Dr. Douglas H. Smith

Department of Neurosurgery, Center for Brain Injury and Repair, University of Pennsylvania, Philadelphia, PA 19104, USA

E-mail: smithdou@pennmedicine.upenn.edu

Address: 3320 Smith Walk, 105 Hayden Hall

Phone: 215-573-3156

Fax: 215-573-3808

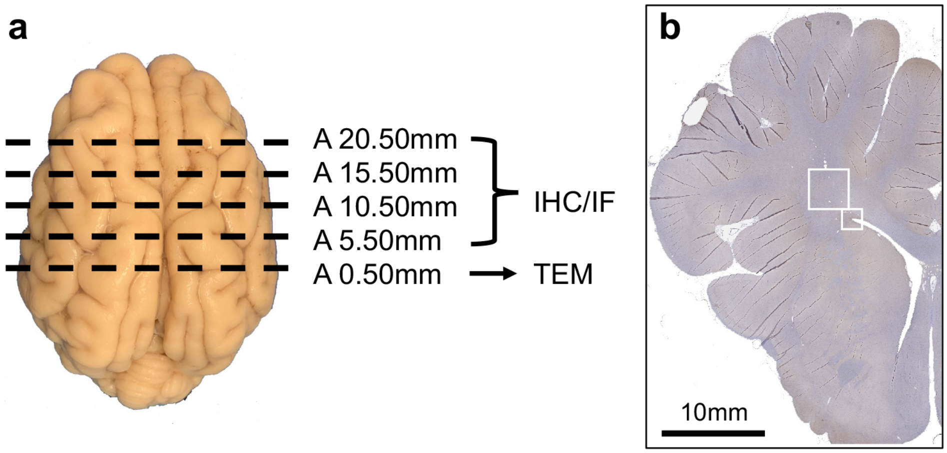

Supplemental Fig. 1. Schemes of swine brain tissue preparation and focused examination of deep white matter and subcallosal fasciculus. (a) Representative swine brain top view shows the levels of brain examined in the coronal plane for IHC and TEM. (b) Scheme from a whole slide image showing the left hemisphere of a brain coronal level (Scale bar = 10mm). Within it, the inset white boxes represent deep white matter and subcallosal fasciculus of IHC measurements for statistical analysis in Fig. 2 of microscopic APP-immunoreactive axonal profiles.

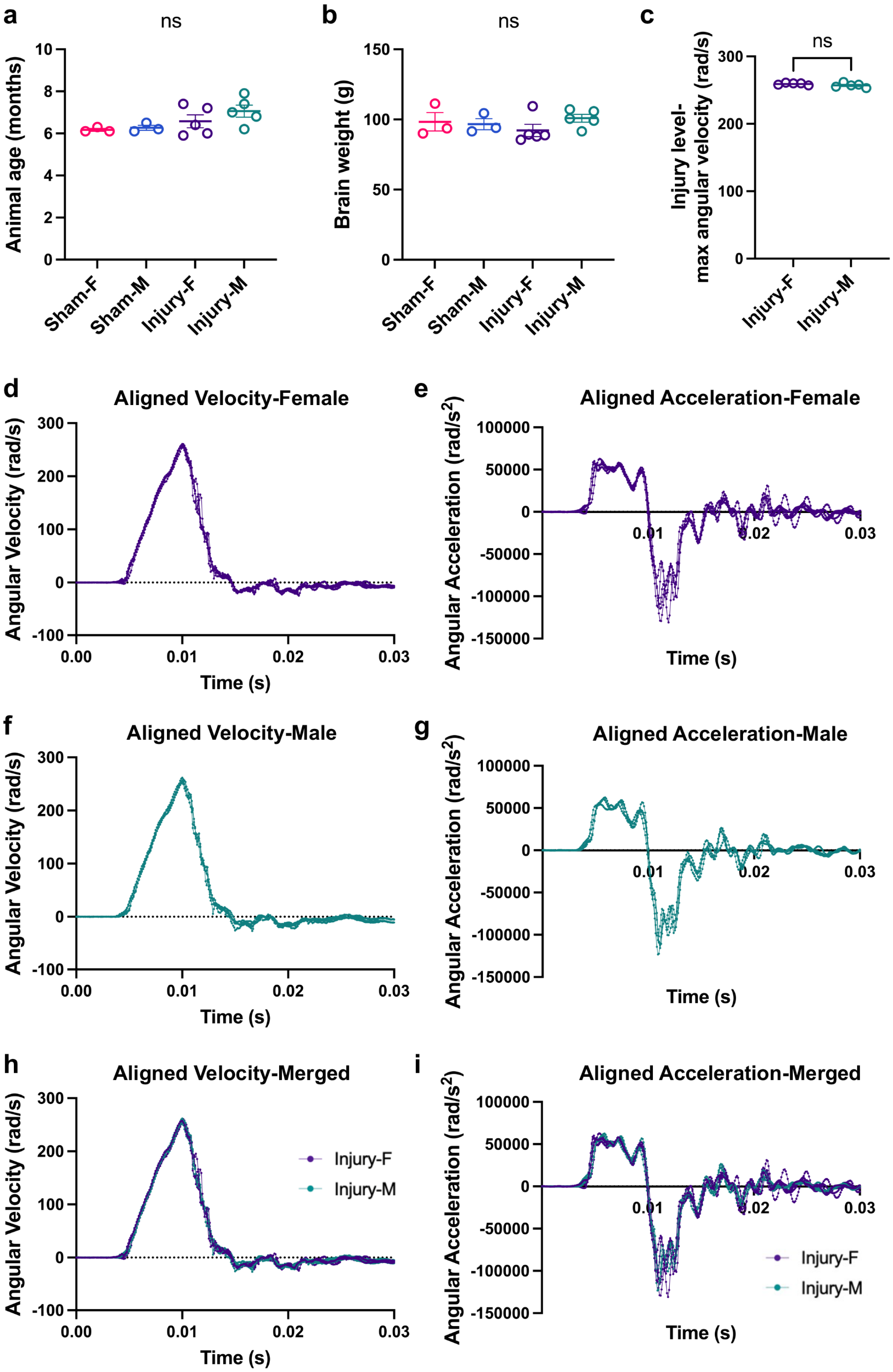
a
ns
Animal age (months)
Sham-F
Sham-M
Injury-F
Injury-M
b
ns
Brain weight (g)
c
ns
Injury level-
max angular velocity (rad/s)
d
Aligned Velocity-Female
Angular Velocity (rad/s)
Time (s)
e
Aligned Acceleration-Female
Angular Acceleration (rad/s^2)
f
Aligned Velocity-Male
g
Aligned Acceleration-Male
h
Aligned Velocity-Merged
i
Aligned Acceleration-Merged

Supplemental Fig. 2. Animal characteristics and kinematic parameters following experimental concussion. (a-b) Female and male swine had no statistical difference in ages and brain weights (‘ns’ indicating not significant). (c) Female and male animals sustained similar levels of head rotational acceleration injury, demonstrated by maximum angular velocity. (d-i) Similarly, no difference was noted in angular velocity and angular acceleration traces, including injury velocity magnitude, acceleration peaks and acceleration durations, between injured female and male animals.

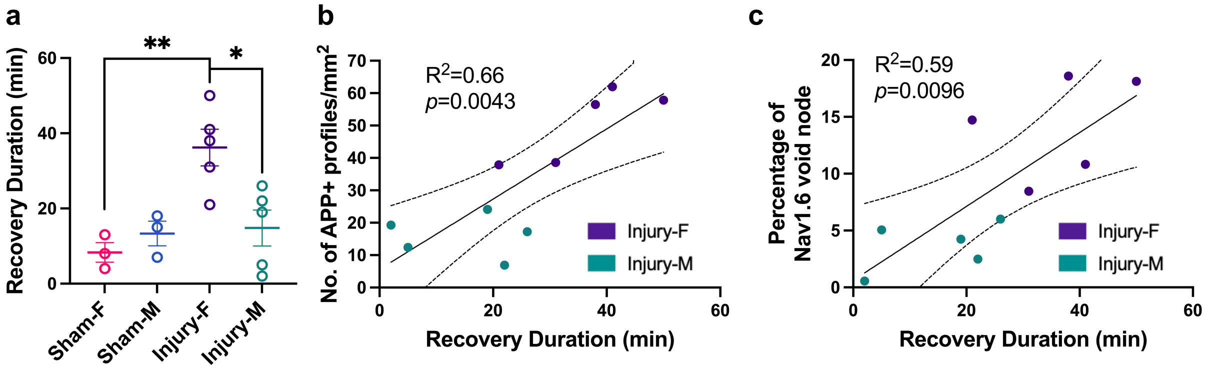

Supplemental Fig. 3. Animal recovery duration and its correlation with the extent of axonal pathologies. (a) Greater recovery duration in females after injury than males. (b) The extent of APP axonal pathology at the level of A 15.50mm was positively correlated with animal recovery duration. Additional comparisons at other levels can be found in Supplemental Table 1. (c) Similarly, overt Nav1.6 loss (percentage of void node) was positively correlated with animal recovery duration.

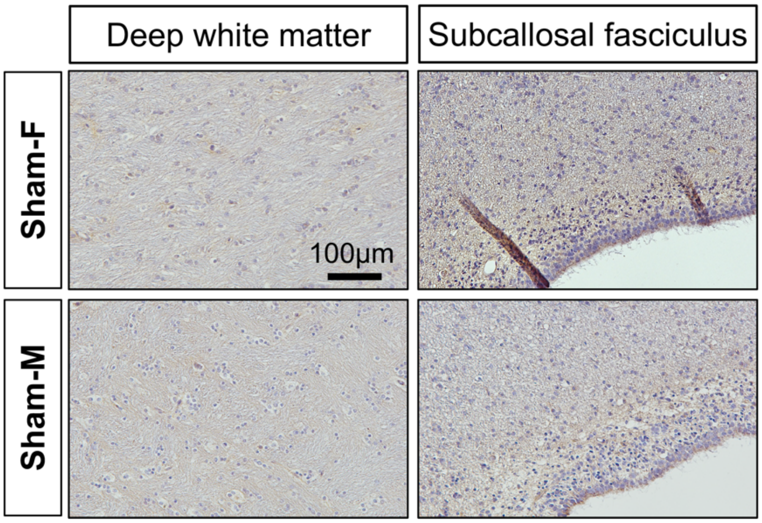

Supplemental Fig. 4. No APP axonal pathology in sham animals. No obvious microscopic APP-immunoreactive profile was observed in both sham female and male swine.

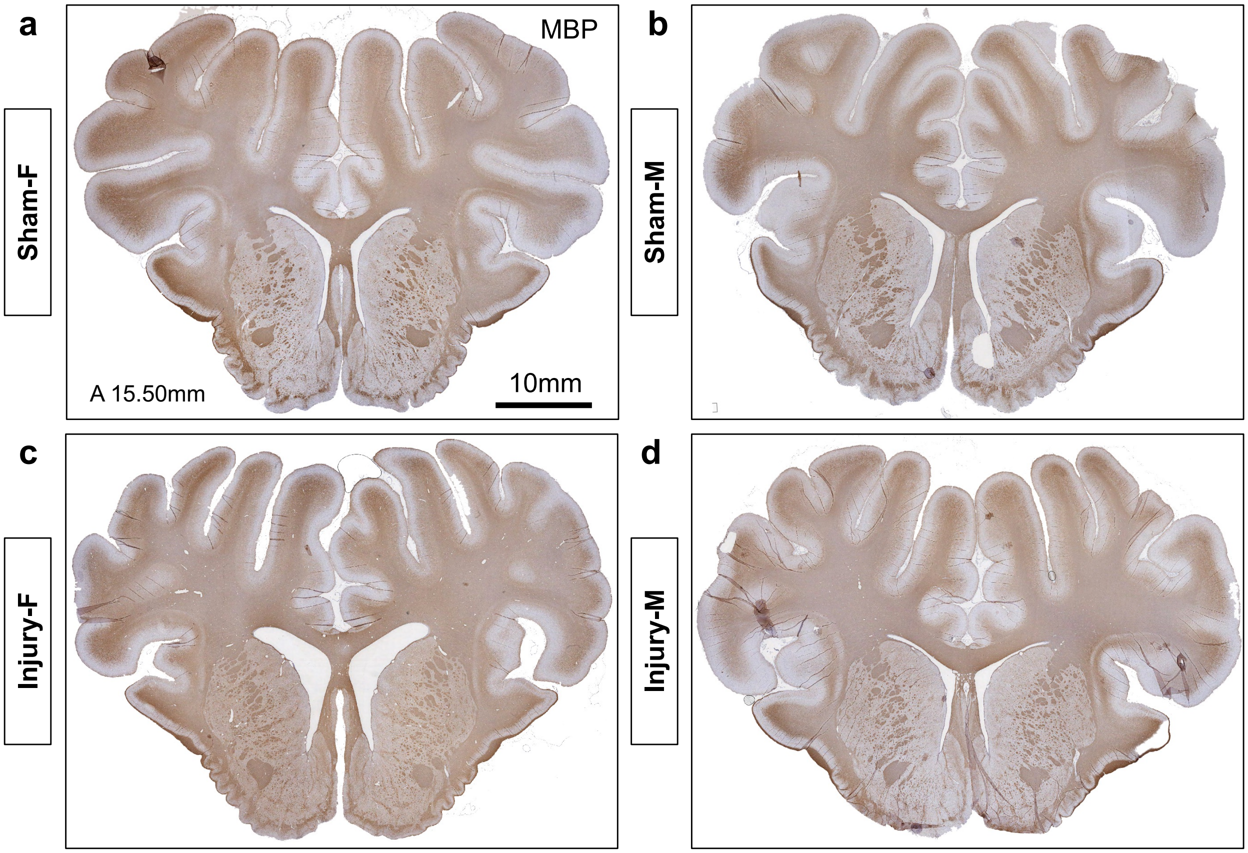

Supplemental Fig. 5. No overt loss of myelin content following concussion. No obvious change in MBP immunoreactivities was observed after injury in both female and male swine.

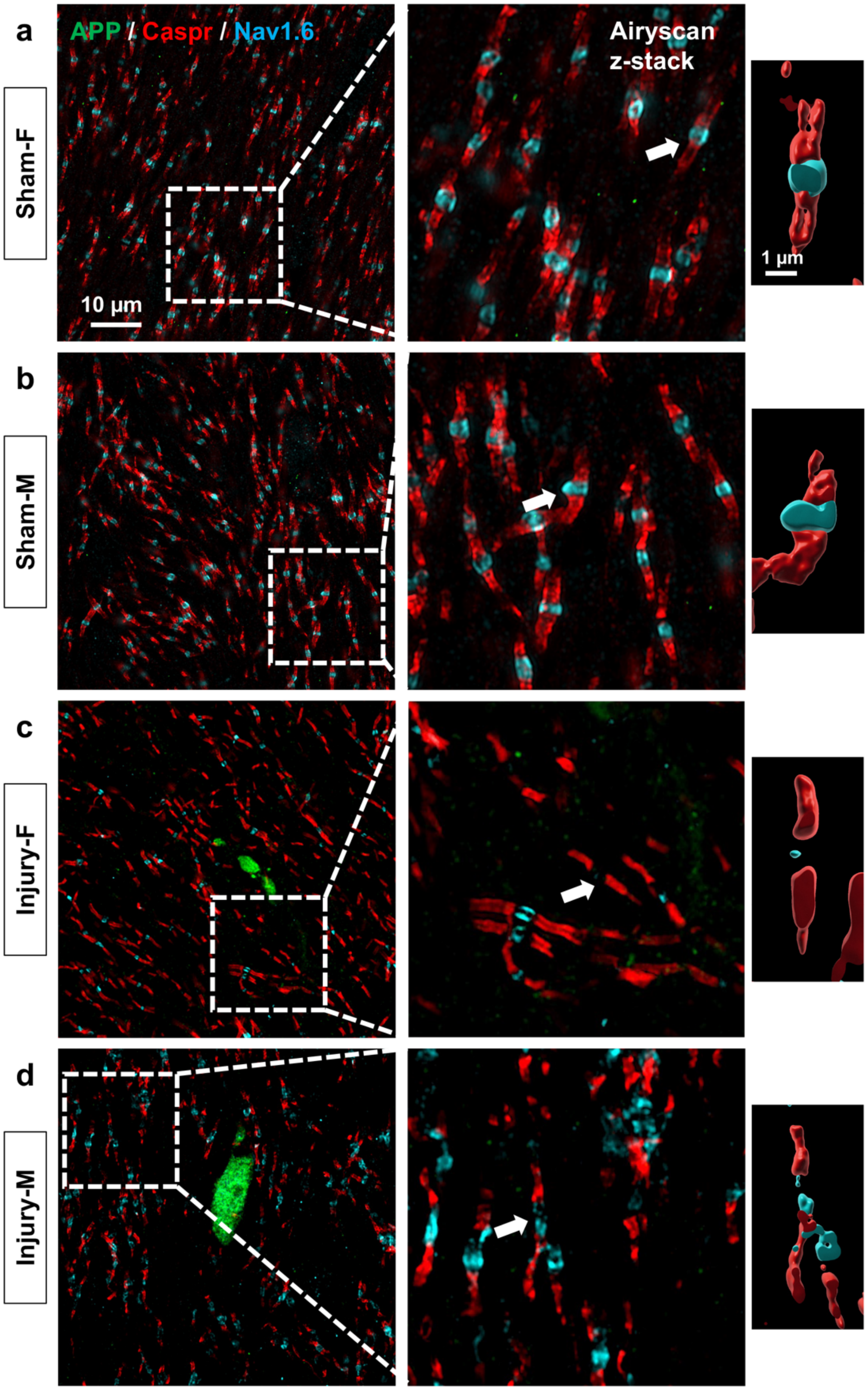

Supplemental Fig. 6. Nav1.6 loss in subcallosal fasciculus following injury. (a-b) Representative images from brain subcallosal fasciculus show normal Nav1.6 expression in both

sham female and male swine. (c-d) At 24 hours post injury, injured females displayed greater loss of Nav1.6 than males. White arrow labels the characteristic Nav1.6 loss from the enlarged view, which is then confirmed by 3D reconstruction.

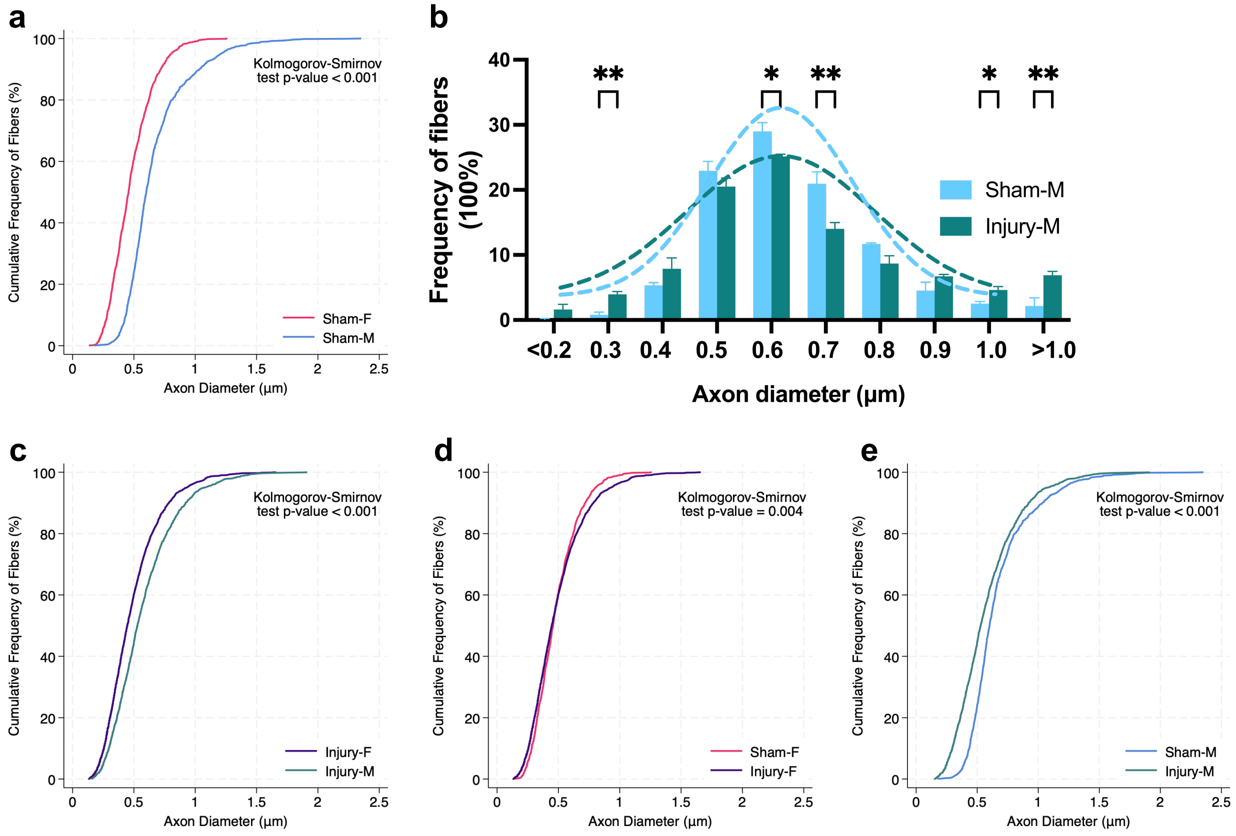

Supplemental Fig. 7. Axon caliber differences and changes between female and male swine. (a) Significant difference in cumulative frequency of axon diameter (μm) between sham female and sham male animals. (b) Histogram of axon fiber frequency shows differences in axon diameter distribution between sham male and injured male axons (best fitting curve in dashed line with Gaussian distribution). (c-e) Significant differences in cumulative frequency of axon caliber between injury female vs. injury male, sham female vs. injury female, and sham male vs. injury male animals.

Supplemental Table. 1. Linear regression statistics on the correlation between recovery duration and the extent of axonal pathologies

Correlation between	Recovery duration and No. of APP profiles within deep white matter at			
	**A 20.50mm level**	**A 15.50mm level**	**A 10.50mm level**	**A 5.50mm level**
Goodness of Fit				
R squared	0.4209	0.6599	0.6736	0.1657
P value	0.0424	0.0043	0.0036	0.2431
Equation	Y = 0.5176*X + 4.571	Y = 1.083*X + 5.654	Y = 0.4905*X + 1.748	Y = 0.1311*X + 5.609
95% Confidence Intervals				
Slope	0.02260 to 1.013	0.4491 to 1.716	0.2122 to 0.7689	-0.1088 to 0.3711
Y-intercept	-9.925 to 19.07	-12.91 to 24.21	-6.404 to 9.900	-1.418 to 12.64
X-intercept	-763.6 to 10.83	-50.49 to 8.030	-43.64 to 8.905	-infinity to 4.228
**Correlation between**	**Recovery duration and No. of APP profiles within subcallosal fasciculus at**			
	**A 20.50mm level**	**A 15.50mm level**	**A 10.50mm level**	**A 5.50mm level**
Goodness of Fit				
R squared	0.3897	0.804	0.3229	0.478
P value	0.0537	0.0004	0.0866	0.0268
Equation	Y = 1.385*X + 51.86	Y = 1.284*X + 84.81	Y = 1.215*X + 46.92	Y = 1.440*X + 24.02
95% Confidence Intervals				
Slope	-0.02821 to 2.799	0.7674 to 1.801	-0.2193 to 2.648	0.2133 to 2.667
Y-intercept	10.46 to 93.26	69.67 to 99.95	4.927 to 88.92	-11.91 to 59.95
X-intercept	-infinity to -3.968	-128.2 to -39.31	-infinity to -1.991	-261.4 to 4.803
**Correlation between**	**Recovery duration and % of Nav1.6 void node at**			
	**Deep white matter**		**Subcallosal fasciculus**	
Goodness of Fit				
R squared	0.5889		0.4807	
P value	0.0096		0.0262	
Equation	Y = 0.3250*X + 0.6219		Y = 0.3079*X + 3.946	
95% Confidence Intervals				
Slope	0.1036 to 0.5464		0.04699 to 0.5688	

Y-intercept	-5.861 to 7.105	-3.695 to 11.59
X-intercept	-62.96 to 11.68	-227.8 to 7.031
